# Co-Occurring Infections in Cancer Patients Treated with Checkpoint Inhibitors Significantly Increase the Risk of Immune-Related Adverse Events

**DOI:** 10.3390/cancers16162820

**Published:** 2024-08-11

**Authors:** Siranuysh Grabska, Hovakim Grabski, Tigran Makunts, Ruben Abagyan

**Affiliations:** 1 Skaggs School of Pharmacy and Pharmaceutical Sciences, University of California San Diego, La Jolla, CA 92093, USA; hgrabski@health.ucsd.edu (H.G.); tmakunts@health.ucsd.edu (T.M.); 2 L.A. Orbeli Institute of Physiology, National Academy of Sciences, Yerevan 0028, Armenia

**Keywords:** immune checkpoint inhibitors, infection, cancer, pembrolizumab, nivolumab, durvalumab, atezolizumab, ipilimumab, myocarditis, colitis

## Abstract

**Simple Summary:**

This study analyzed over eighty thousand adverse event reports from the Food and Drug Administration’s Adverse Event Reporting System to identify records of patients administered with immune checkpoint inhibitors as monotherapy for various malignancies. We analyzed whether co-occurring viral, bacterial, or fungal infections increased the risk of developing immune-related adverse events, such as pneumonitis, sarcoidosis, myocarditis, nephritis, colitis, hepatitis, and others, in cancer patients treated with those inhibitors and quantified the association. We have found a statistically significant, at the 95% confidence level, association between co-occurring infections and immune-related adverse events in cancer patients treated with immune checkpoint inhibitors.

**Abstract:**

Therapeutic antibodies designed to target three immune checkpoint proteins have been applied in the treatment of various malignancies, including small and non-small cell lung cancers, melanoma, renal cell carcinoma, and others. These treatments combat cancers by reactivating cytotoxic T cells. Nevertheless, this mode of action was found to be associated with a broad range of immune-related adverse events (irAEs), including pneumonitis, sarcoidosis, myocarditis, nephritis, colitis, and hepatitis. Depending on their severity, these irAEs often necessitate the suspension or discontinuation of treatment and, in rare instances, may lead to fatalities. We analyzed over nineteen million reports and identified over eighty thousand adverse event reports from patients treated with immune checkpoint inhibitors submitted to the Food and Drug Administration’s Adverse Event Reporting System MedWatch. Reports concerning pembrolizumab, nivolumab, cemiplimab, avelumab, durvalumab, atezolizumab, and ipilimumab revealed a statistically significant association between the irAEs and concurrent infectious diseases for five out of seven treatments. Furthermore, the association trend was preserved across all three types of checkpoint inhibitors and each of the five individual therapeutic agent cohorts, while the remaining two showed the same trend, but an increased confidence interval, due to an insufficient number of records.

## 1. Introduction

Cancer immunotherapy, a real step forward in cancer treatment, has gained recognition due to the success of targeted immune checkpoint inhibitors (ICIs) in the treatment of a wide range of malignancies, including non-small cell lung cancers, melanoma, and renal cell carcinoma. The breakthrough was achieved by reactivation of the host immune system, instead of aiming only at the targets in cancer cells [1]. These treatments combat cancers by reactivating CD8 cytotoxic T cells [2,3,4]. In 2011, the first immunotherapy antibody, ipilimumab (Yervoy) [5], which blocks the cytotoxic T-lymphocyte-associated antigen 4 (CTLA-4), was approved [5]. Antibodies targeting the programmed cell death protein 1 (PD-1) receptors, pembrolizumab (Keytruda), nivolumab (Opdivo), and cemiplimab (Libtayo), as well as antibodies targeting PD-1 ligand (PD-L1), including atezolizumab (Tecentriq), durvalumab (Imfinzi), and avelumab (Bavencio) [2,6], were approved later. 

Treatment with checkpoint inhibitors has been associated with significant immune-related adverse events (abbreviated as irAEs) [7]. Inhibiting immune checkpoints can lead to the activation of auto-reactive T cells and consequently result in various irAEs impacting organ systems, including the pulmonary (e.g., pneumonitis), cardiac (e.g., myocarditis, pericarditis), renal (e.g., nephritis), gastrointestinal (e.g., colitis), and hepatic (e.g., hepatitis) [7] systems. Considering the concern about severe immune side effects from ICIs, the question arises as to whether an occasional infectious disease could exacerbate these side effects to potentially dangerous levels.

In a prior study of data from the KEYNOTE clinical trial of pembrolizumab [8,9,10], we observed a statistically and clinically significant association between co-occurring infections and irAEs [11]. This finding warranted extending the pembrolizumab study to all clinically used ICIs. United States Food and Drug Administration (FDA) Adverse Event Reporting System (FAERS) post-marketing surveillance data were used as the data source. Given the differences in the mechanisms of action of the ICIs, it was not clear if the trend observed for pembrolizumab would remain for other ICIs, considering their different targets and individual properties. Here, we studied the occurrence of immune-related adverse effects, including pneumonitis, sarcoidosis, myocarditis, pericarditis, nephritis, colitis, and hepatitis, while receiving an immune checkpoint inhibitor treatment, in cancer patients with or without a co-occurring infection to quantify the risk of irAEs associated with different types of treatments.

## 2. Materials and Methods

### 2.1. FDA Adverse Event Reporting System and MedWatch

FAERS, including its original version AERS, is a database of post-marketing safety surveillance reports, and it operates under the auspices of the FDA. Reporting of adverse events (AEs) and their associated outcomes to FAERS primarily occurs through the MedWatch system (forms 3500 and 3500A) [12]. It includes voluntary submissions from physicians, pharmacists, consumers, legal representatives, nurses, and other healthcare professionals. Reports first submitted to the sponsor, vendor, or manufacturer are required by regulations to be deposited in FAERS too. 

At the time of the analysis, the FAERS dataset consisted of over nineteen million six hundred thousand adverse event reports spanning from the first quarter of 2004 (which included reports from the 1990s) to the second quarter of 2023. These reports formed the basis for a retrospective analysis of a subset of monotherapy treatments with immune checkpoint inhibitors.

For online access to FAERS datasets, use the following link: 

https://fis.fda.gov/extensions/FPD-QDE-FAERS/FPD-QDE-FAERS.html (accessed on 20 October 2023) 

All methods and procedures for data analysis adhered to the established guidelines and regulations. Since this study relied solely on publicly available data and the FDA datasets analyzed had been thoroughly reviewed and de-identified prior to release, no further approval from institutional or licensing committees was required.

### 2.2. Data Preparation

The FAERS database consolidates reports from the United States and over 200 countries, each with distinct demographic formats and names of medications. Prior to the initiation of data collection and analysis, online drug databases including Drugs@FDA [13], ChEMBL [14], ZINC [15], PubChem [16], DrugBank [17], and National Library of Medicine [18] were employed to establish a comprehensive dictionary of generic and brand names of drugs to facilitate their translation into unique generic names. Each of the quarterly report datasets from FAERS consists of seven files containing common report IDs. To enhance uniformity across the datasets, each quarterly report set was downloaded in dollar-separated text format and reformatted into combined reports extended with generic names. To create a standardized data table, missing fields in the older FAERS datasets were introduced without specific values [19,20,21]. The resulting comprehensive table contained 19,609,804 adverse event reports. Duplicate records (about 0.4%) were removed prior to the analysis.

### 2.3. Cohort Choice and Study Outcomes 

Prior to analysis, the FAERS database was queried for all the reports of approved PD-1, PD-L1, and CTLA-4 ICIs (*n* = 233,915). Only reports with a single treatment, referred to as *monotherapy* reports, were selected (*n* = 80,927) and further separated into individual classes: PD-1 inhibitors (pembrolizumab, *n* = 22,580; nivolumab, *n* = 38,218; cemiplimab, *n* = 680), PD-L1 inhibitors (avelumab, *n* = 912; durvalumab, *n* = 4398; atezolizumab, *n* = 5310), and CTLA-4 inhibitors (ipilimumab, *n* = 8829). Each ICI cohort was further split into cases with and without co-occurring infections by the preferred query terms specified below. The monotherapy selection was performed to rule out patients with pre-existing immune conditions or infectious diseases that would have required a different treatment. 

Immune-related adverse effect terms were selected from over twenty thousand unique terms listed in FAERS. The following FDA Medical Queries (FMQs) were used to generate a list of preferred term codes to define the infection (primary) and no-infection cohorts with viral infectious disorders (680 terms), bacterial infectious disorders (1267), fungal infections (235), opportunistic infections (172), and renal and urinary infections (100) by searching related terms in reports in each monotherapy-treated patient. A total of 2454 infection codes and related FMQ preferred term (PT) codes (see Data Availability Statement section) [11,22] were used in this study. 

### 2.4. Study Outcomes

The primary measured study outcome was the ratio in reported irAE frequencies for both groups (infection vs. no infection) for each treatment. The irAE PT codes were based on system organ class AE terms of inflammatory nature. The reports indicating infection-related inflammatory conditions (e.g., viral hepatitis, infectious colitis) were excluded from the irAE list due to their unrelated etiology/root cause. However, the immune-related AEs characteristic of enhanced autoimmune reactions were retained. Reporting odds ratio (ROR) disproportionality analysis and relative risk (RR) calculations were performed using their reported irAE counts in each cohort and evaluating the 95% confidence intervals (CIs) of the ROR values (see details in Section 2.5).

### 2.5. Statistical Analysis

#### 2.5.1. Descriptive Statistics

Frequencies for each AE PT code were calculated by the following equation:

$$Frequency\left(\%\right)\;=\;100\%\cdot \frac{n_{AE}}{n}$$

where *n_AE_* is the number of reports with irAEs in a cohort, and *n* is the total number of reports in the cohort.

#### 2.5.2. Comparative Statistics Odds Ratio and Relative Risks

AE report rates were compared via the reporting odds ratio and relative risk analysis using the following equations:

$$ROR=\frac{ad}{bc},\;\mathrm{and}\;RR\;=\frac{a\left(c+d\right)}{c\left(a+b\right)}$$

where *a* is the number of irAE cases in co-occurring infection group; *b* is the number of no irAE cases in the co-occurring infection group; *c* is the number of irAE cases in the control group; and *d* is the number of no irAE cases in the control group.

The standard error of log reporting odds ratio and log relative risks is as follows:

$$SE\left\{\ln\left(ROR\right)\right\}=\sqrt{\frac{1}{a}+\frac{1}{b}+\frac{1}{c}+\frac{1}{d}},\;\mathrm{and}\;SE\left\{\ln\left(RR\right)\right\}=\sqrt{\frac{b}{a\left(a+b\right)}+\frac{d}{c\left(c+d\right)}},$$

95% confidence interval:

$$95\%\;CI=e^{\left(\ln\left(ROR\right)-1.96\times SE\left\{\ln\left(ROR\right)\right\}\right)}\;\mathrm{to}\;e^{\left(\ln\left(ROR\right)+1.96\times SE\left\{\ln\left(ROR\right)\right\}\right)}$$

$$95\%\;CI=e^{\left(\ln\left(RR\right)-1.96\times SE\left\{\ln\left(RR\right)\right\}\right)}\;\mathrm{to}\;e^{\left(\ln\left(RR\right)+1.96\times SE\left\{\ln\left(RR\right)\right\}\right)}$$

## 3. Results

### 3.1. Immune Adverse Effects from the Cancer Treatment Increase with Co-Occurring Infections

Monotherapy reports for each of the seven studied immune checkpoint inhibitors contained a substantial fraction of reported irAEs: pembrolizumab (5270 out of 22,580, 23.3%), nivolumab (7267 out of 38,218, 19.0%), cemiplimab (113 out of 680, 16.6%), avelumab (99 out of 912, 10.9%), durvalumab (1308 out of 4398, 29.7%), atezolizumab (757 out of 5310, 14.3%), and ipilimumab (2160 out of 8829, 24.5%). Surprisingly, the irAE report occurrence in the ipilimumab cohort (CTLA-4-targeting antibody) did not differ significantly from the PD-1 and PD-L1 cohorts (PD-1 total 20.6%, PD-L1 total 20.4% vs. CTLA-4 total 24.5%). 

**Figure 1:**
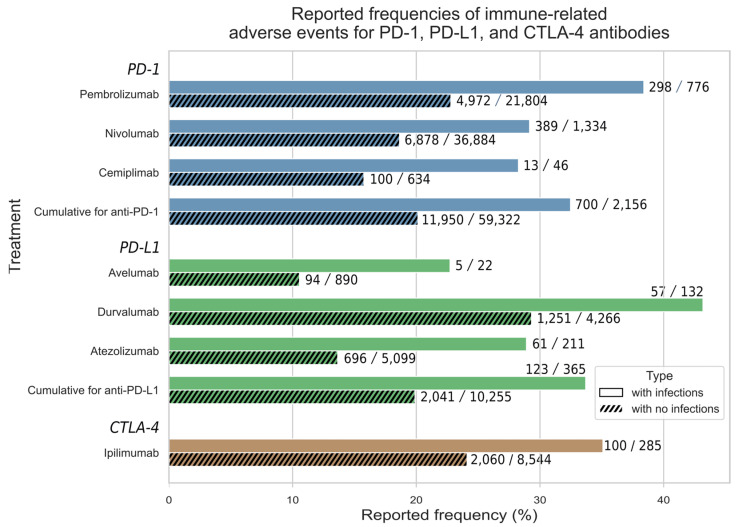
FAERS frequencies of irAE reports in patients administered ICIs with and without co-occurring infections. The number of irAE-containing reports for each sub-cohort is shown along with the total number of reports in that sub-cohort. The bars (X-axis) show the frequencies of irAEs in two cohorts for each of the seven drugs, and in two combined PD-1 and PD-L1 groups. The fraction of the infected patients for each treatment were as follows: pembrolizumab 3.44%, nivolumab 3.49%, cemiplimab 6.76%, avelumab 2.41%, durvalumab 3%, atezolizumab 3.97%, ipilimumab 3.23%.

Each individual monotherapy cohort was split into co-occurring infection and no-co-occurring infection sub-cohorts, and the reported irAE frequencies were compared (Figure 1).

The sub-cohorts of patients with co-occurring infections experienced a higher rate of immune side effects for all the drugs studied. In comparison, the no-infection sub-cohorts had immune-related adverse event (irAE) rates ranging from 11% to 29%, whereas those with infections had rates between 22% and 43%. This increase was consistently observed across each monotherapy, as further detailed in the next section. Furthermore, the differences in reported irAE frequencies between infected and uninfected sub-cohorts, for each ICI class and individual ICI cohorts with more than one thousand reports, were statistically significant across all the ICI class cohorts (see Figure 1). 

### 3.2. Reporting Odds Ratios and Relative Risks Are Significant for All Drugs with over a Thousand Reports

**Figure 2:**
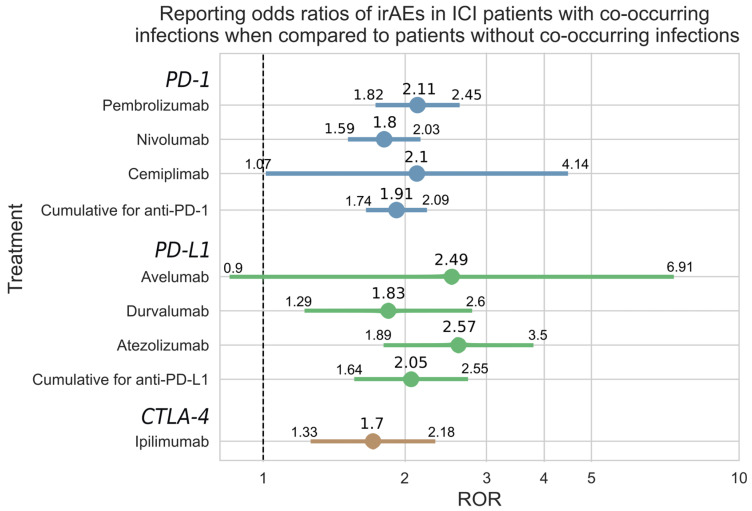
Reporting odds ratios (RORs) of irAEs in ICI patients with and without co-occurring infections. The depicted ranges represent 95% confidence intervals (see Section 2). The X-axis shows odds ratios (circle) and their confidence intervals (horizontal bar) on a logarithmic scale.

Patients administered ICIs who had a co-occurring infection had a higher risk of developing irAEs according to the reporting odds ratios and 95% confidence intervals (CIs; see Section 2): pembrolizumab ROR 2.11 (95% CI [1.82, 2.45]), nivolumab 1.80 (1.59, 2.03), cemiplimab 2.10 (1.07, 4.14), avelumab 2.49 (0.90, 6.91), durvalumab 1.83 (1.29, 2.60), atezolizumab 2.57 (1.89, 3.50), and ipilimumab 1.70 (1.33, 2.18). For avelumab, although the trend was preserved, the risk was not statistically significant: 2.49 (0.90, 6.91). Cemiplimab has a borderline lower bound value of 1.07, which technically means that it is statistically significant according to the accepted 95% CI definition. Additionally, when analyzed cumulatively for all three ICI classes, there was a statistically increased risk of ICIs when a co-occurring infection was present: PD-1 inhibitors 1.91 (1.74, 2.09), PD-L1 inhibitors 2.05 (1.64, 2.55), CTLA-4 inhibitor (ipilimumab) 1.70 (1.33, 2.18) (Figure 2).

**Figure A1:**
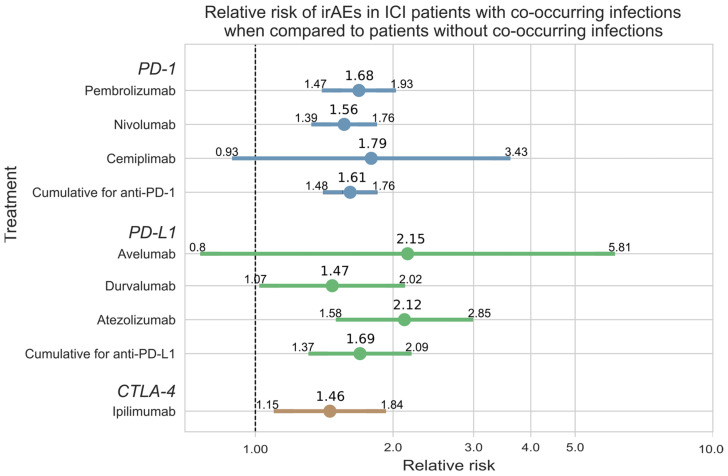
Relative risk (RR) of irAEs in ICI patients with and without co-occurring infections. The depicted ranges represent 95% confidence intervals (see Section 2). The X-axis shows relative risk (circle) and their confidence intervals (horizontal bar) on a logarithmic scale.

An increased incidence of immune-related adverse events for infected patients receiving cancer immunotherapy was also observed when calculating the relative risk and confidence intervals (CIs; see Section 2): pembrolizumab 1.68 (95% CI [1.47, 1.93]), nivolumab 1.56 (1.39, 1.76), cemiplimab 1.79 (0.93, 3.43), avelumab 2.15 (0.80, 5.81), durvalumab 1.47 (1.07, 2.02), atezolizumab 2.12 (1.58, 2.85), and ipilimumab 1.46 (1.15, 1.84). In the case of relative risk for cemiplimab and avelumab, while the trend was consistent, statistical significance was not reached (1.79 (0.93, 3.43) and 2.15 (0.80, 5.81)) due to an insufficient number of observations. For each of the three ICI drug groups, the RR values were significant: PD-1 inhibitors (1.61 (1.48, 1.76)), PD-L1 inhibitors (1.69 (1.37 2.09)), and CTLA-4 inhibitor ipilimumab (1.46 (1.15, 1.84), Figure A1).

## 4. Discussion

In this study, we analyzed over eighty thousand FAERS adverse event reports for individuals treated with seven approved immune checkpoint inhibitors as monotherapy for various malignancies. Our concern was related to the immune-related adverse events, such as immune-mediated pneumonitis, colitis, hepatitis, endocrinopathies, nephritis, or many other immune system problems, listed as side effect warnings for the ICIs. The concern was that these rare but serious side effects may become more frequent with a co-occurring infection. These adverse reactions can occur in any organ system or tissue and can be severe or fatal. To the best of our knowledge, this is the first comprehensive examination of concurrent irAEs in subjects with and without co-occurring infections using population-scale post-marketing data. The fraction of the co-infected patients for all treatments was 2.4% for avelumab to 6.8% for cemiplimab, both with an insufficient number of records, while the infected fractions for the main five monotherapy cohorts were 3% to 4%. In this paper, we did not analyze whether the observed fractions were elevated due to the treatment.

The analysis revealed that the risk of experiencing an immune-related adverse event increased by 80 to 160% in cases where individuals experienced a co-occurring infection during their treatment with immune checkpoint inhibitors. However, the relationship between the irAEs and the co-occurring infections was complex, with no clear indication of causality due to the absence of temporal details in the reports. This lack of longitudinal data made determining causality challenging. Nevertheless, the association was statistically significant and may have clinical implications for cancer patients at higher risk of immune-related complications. It was expected that infectious diseases lead to additional immune response; here, we simply quantified this phenomenon. 

Previously, a few case reports and smaller-scale studies have discussed the connection between irAEs and infections [23,24], and some authors attributed related organ damage to irAE exacerbation during concurrent infections [25]. However, this association had not been quantified and statistically evaluated in large-scale studies. Although it seems intuitive that an infection could impact irAEs, the extent of this association had not been previously assessed. In terms of the molecular and physiological mechanism of this association, some studies linked infections with autoimmune diseases (ADs), which share similarities with irAEs in their manifestation, physiological profile, and molecular mechanisms involving the innate and adaptive immune systems, including arthritis, autoimmune thyroiditis, colitis, and lupus [26,27,28,29]. In the latter cases, infection-related T-cell autoreactivity is the primary culprit. Mechanisms through which infectious agents might cause irAEs include cryptic antigen presentation, bystander activation, molecular mimicry, and epitope spreading [30].

Finally, the observed increase in immune-related adverse effects in patients with co-occurring infections calls for more careful consideration of the ICI treatment scheduling and dosing, as well as monitoring of those patients for the signs and symptoms of a range of adverse effects and related toxicities in all organ systems implicated in irAEs.

## 5. Conclusions

In summary, we observed a statistically significant association between co-occurring infections and immune-related adverse events in cancer patients treated with PD-1, PD-L1, or CTLA-4 inhibitors. Our findings highlight that this association is not only present but robust, as demonstrated by the statistically significant reporting odds ratios and their confidence intervals. These findings suggest that careful monitoring for infections in patients undergoing such immunotherapies may be crucial to mitigate potential adverse effects and improve patient outcomes.

## 6. Study Limitations

The causality between infections and irAEs was not clinically adjudicated due to the lack of comprehensive medical and laboratory records. However, the use of population-scale post-marketing surveillance data provides a robust signal that may have clinical significance. Although we examined the data for the lack of concomitant medications and medical history to rule out potential confounders, it is important to note that consumers and healthcare professionals often under-report over-the-counter medications, supplements, and even prescribed medications. Additionally, non-clinically significant medical events such as minor infections may go unreported, introducing noise or uncertainties to the analysis due to the possible induction of autoimmunity by these factors [31,32]. IrAEs often go under-reported due to the complexities of precise diagnosis, which may require an invasive procedure, often leading to mischaracterizations and misattribution of these adverse events.

## Data Availability

The datasets analyzed for this study can be found in the FAERS database. For online access to FAERS/AERS datasets, please refer to the following link: https://fis.fda.gov/extensions/FPD-QDE-FAERS/FPD-QDE-FAERS.html. The FDA Medical Queries tables were made available online in September 2022 and may be accessed at https://downloads.regulations.gov/FDA-2022-N-1961-0001/attachment_1.xlsm.

## Abbreviations

AE	adverse event
AERS	adverse event reporting system
AD	autoimmune disease
CI	confidence interval
CTLA-4	cytotoxic T-lymphocyte-associated antigen 4
FDA	Food and Drug Administration
FAERS	FDA Adverse Event Reporting System
FMQs	FDA Medical Queries
ICI	immune checkpoint inhibitors
irAE	immune-related adverse events
PD-1	programmed cell death protein 1
PD-L1	programmed cell death protein 1 ligand
ROR	reporting odds ratios
PT	preferred term
